# Using Twitter-Based Data for Sexual Violence Research: Scoping Review

**DOI:** 10.2196/46084

**Published:** 2023-05-15

**Authors:** Jia Xue, Bolun Zhang, Qiaoru Zhang, Ran Hu, Jielin Jiang, Nian Liu, Yingdong Peng, Ziqian Li, Judith Logan

**Affiliations:** 1 Factor-Inwentash Faculty of Social Work University of Toronto Toronto, ON Canada; 2 Faculty of Information University of Toronto Toronto, ON Canada; 3 Faculty of Arts and Science University of Toronto Toronto, ON Canada; 4 Department of Medicine Center for Gender & Sexual Health Equity University of British Columbia Vancouver, BC Canada; 5 John P Robarts Library University of Toronto Toronto, ON Canada

**Keywords:** Twitter data, sexual violence, sexual assault, scoping review, review method, data analysis, data collection, Twitter, social media, women’s health, violence, abuse, public health, domestic violence

## Abstract

**Background:**

Scholars have used data from in-person interviews, administrative systems, and surveys for sexual violence research. Using Twitter as a data source for examining the nature of sexual violence is a relatively new and underexplored area of study.

**Objective:**

We aimed to perform a scoping review of the current literature on using Twitter data for researching sexual violence, elaborate on the validity of the methods, and discuss the implications and limitations of existing studies.

**Methods:**

We performed a literature search in the following 6 databases: APA PsycInfo (Ovid), Scopus, PubMed, International Bibliography of Social Sciences (ProQuest), Criminal Justice Abstracts (EBSCO), and Communications Abstracts (EBSCO), in April 2022. The initial search identified 3759 articles that were imported into Covidence. Seven independent reviewers screened these articles following 2 steps: (1) title and abstract screening, and (2) full-text screening. The inclusion criteria were as follows: (1) empirical research, (2) focus on sexual violence, (3) analysis of Twitter data (ie, tweets or Twitter metadata), and (4) text in English. Finally, we selected 121 articles that met the inclusion criteria and coded these articles.

**Results:**

We coded and presented the 121 articles using Twitter-based data for sexual violence research. About 70% (89/121, 73.6%) of the articles were published in peer-reviewed journals after 2018. The reviewed articles collectively analyzed about 79.6 million tweets. The primary approaches to using Twitter as a data source were content text analysis (112/121, 92.5%) and sentiment analysis (31/121, 25.6%). Hashtags (103/121, 85.1%) were the most prominent metadata feature, followed by tweet time and date, retweets, replies, URLs, and geotags. More than a third of the articles (51/121, 42.1%) used the application programming interface to collect Twitter data. Data analyses included qualitative thematic analysis, machine learning (eg, sentiment analysis, supervised machine learning, unsupervised machine learning, and social network analysis), and quantitative analysis. Only 10.7% (13/121) of the studies discussed ethical considerations.

**Conclusions:**

We described the current state of using Twitter data for sexual violence research, developed a new taxonomy describing Twitter as a data source, and evaluated the methodologies. Research recommendations include the following: development of methods for data collection and analysis, in-depth discussions about ethical norms, exploration of specific aspects of sexual violence on Twitter, examination of tweets in multiple languages, and decontextualization of Twitter data. This review demonstrates the potential of using Twitter data in sexual violence research.

## Introduction

### Background

Sexual violence is a global public health problem, threatening individuals’ health and well-being worldwide [1]. It is defined as “any sexual act, attempt to obtain a sexual act, unwanted sexual comments or advances, or acts to traffic, or otherwise directed, against a person’s sexuality using coercion, by any person regardless of their relationship to the victim, in any setting, including but not limited to home and work” [2]. Sexual violence victimization can lead to several adverse health outcomes, including self-perceived poor health [3], sexually transmitted infections (eg, HIV), unintended pregnancies, and mental health issues, such as depression, suicide attempts, and posttraumatic stress disorder [4].

Twitter, launched in 2006, has become one of the most widely used social media platforms and an important source of public health information [5-8] and the COVID-19 pandemic [9,10]. Over the past decade, researchers have conducted numerous studies using Twitter to gain insights into sexual violence. Twitter has become a significant venue for political protest [11], the feminist movement [12,13], digital feminist activism [14], and agenda setting for partner violence [15]. The MeToo campaign has been crucial in facilitating public disclosure of sexual assault experiences on social media, including Twitter. Survivors of sexual violence have used Twitter to disclose and share their experiences and connect with other survivors who have had similar experiences. This has enabled survivors to gain a deeper understanding of their experiences and has been seen as an essential step in the healing process [16]. Twitter has been used extensively to discuss the MeToo campaign [11,17,18] and other related hashtags such as #NotOkay [19], #UsToo [20], #WhyIStayed [21], and #WhyILeft [22].

Research has also explored social reactions to sexual violence disclosures on Twitter, with the use of hashtags such as #NotOkay [11]. There have also been debates regarding high-profile cases of sexual violence, such as the Janay Rice and Ray Rice case [23], as well as reactions to disclosures of sexual violence in the sports context [24]. Researchers have also analyzed hidden topics and thematic structures of sexual violence texts on Twitter [25]. The public response to disclosures of sexual violence can provide valuable insights to researchers on how bystanders react to such disclosures and help develop social media–based bystander programs. Negative social reactions to disclosures of sexual violence may dissuade survivors from disclosing their experiences, thereby making it difficult for them to access online support [26]. Sexual assault survivors have used Twitter to seek health information or build online communities where they can discuss the magnitude of the social problem. Using network analysis, researchers have also explored how topics related to sexual violence are posted and disseminated on Twitter [25].

In the digital age, Twitter has emerged as a crucial platform for understanding sexual violence. Understanding how the Twitter data set can be harnessed is essential to contribute to our understanding of sexual violence research. Therefore, exploring the ways in which Twitter data can be effectively used in sexual violence research can provide valuable insights to practitioners and researchers to effectively use the platform’s potential for implementing violence prevention and intervention strategies.

### Aim of the Study

This study aims to provide a scoping review of the current literature on using Twitter data for sexual violence research. This review focuses primarily on the current state of Twitter data in sexual violence research, including (1) describing the main objectives addressed by previous studies in the field; (2) evaluating the methodology of these studies, including aspects such as study design, data collection, and data analysis strategies; (3) presenting a taxonomy describing the ways in which Twitter is used as a data source in sexual violence research; (4) presenting the various ways in which Twitter metadata features are used in this regard; and (5) discussing the implications and limitations of using Twitter data for sexual violence research. By providing a scoping overview of the existing research, this study contributes to a better understanding of the potential of Twitter data for sexual violence research and provides insights into the evidence-based practices for conducting such research.

## Methods

### Overview

This study was guided by Arksey and O’Malley’s scoping review framework [24]. This study was carried out in five stages, including (1) identifying research questions; (2) identifying relevant studies; (3) selecting studies; (4) charting the data; and (5) collating, summarizing, and reporting the results [27].

### Identifying Relevant Studies

A social and health sciences librarian (JL) developed a search strategy for this review. The librarian drafted an initial search strategy validated against a predetermined test set of articles and peer-reviewed by an independent librarian colleague using the Peer Review of Electronic Search Strategies (PRESS) framework [28]. This search was structured to retrieve articles examining “sexual violence and Twitter” or “hashtags specific to sexual violence,” such as #MeToo and #TimesUp, using both text words and subject headings. We used a test set of 13 predetermined studies to validate the primary search strategy in APA PsycInfo (Ovid). We iteratively refined the initial search strategy to ensure all 13 studies would pass the initial screening. Once finalized, the search strategy was translated into 5 other databases to locate relevant peer-reviewed studies without geographical restrictions. In total, we searched six databases, including (1) APA PsycInfo (Ovid), (2) Scopus, (3) PubMed, (4) International Bibliography of Social Sciences (ProQuest), (5) Criminal Justice Abstracts (EBSCO), and (6) Communication Abstracts (EBSCO). Results were filtered by date starting from 2006, the year of Twitter’s inception. No other filters were applied. These searches were last run on April 14, 2022, and uploaded to Covidence for deduplication and screening. The search strategy is available in Multimedia Appendix 1.

### Selecting Studies

#### Inclusion and Exclusion Criteria

To be included in the review, studies had to meet the following criteria: (1) be empirical research, (2) focus on sexual violence (eg, if the study just mentioned the term “sexual violence” or “sexual assault,” it was not eligible), (3) analyze Twitter data (ie, tweets or Twitter metadata), and (4) be written in English. In this review, we defined sexual violence as “a sexual act that is committed or attempted by another person without freely given consent of the victim or against someone unable to consent or refuse” [29]. Aligning with this definition, we included various terms commonly used in the literature to describe sexual violence, such as rape, sexual assault, sexual abuse, sexual harassment, sexual coercion, and sexual aggression. Studies were excluded from the review if (1) Twitter data were not analyzed in the study, for example, Twitter was used merely as a platform to recruit participants, and the sample consisted of human beings rather than Twitter data; (2) studies were nonempirical research (eg, reviews and comments); or (3) studies were written in languages other than English.

#### Selection Procedure and Search Results

**Figure 1:**
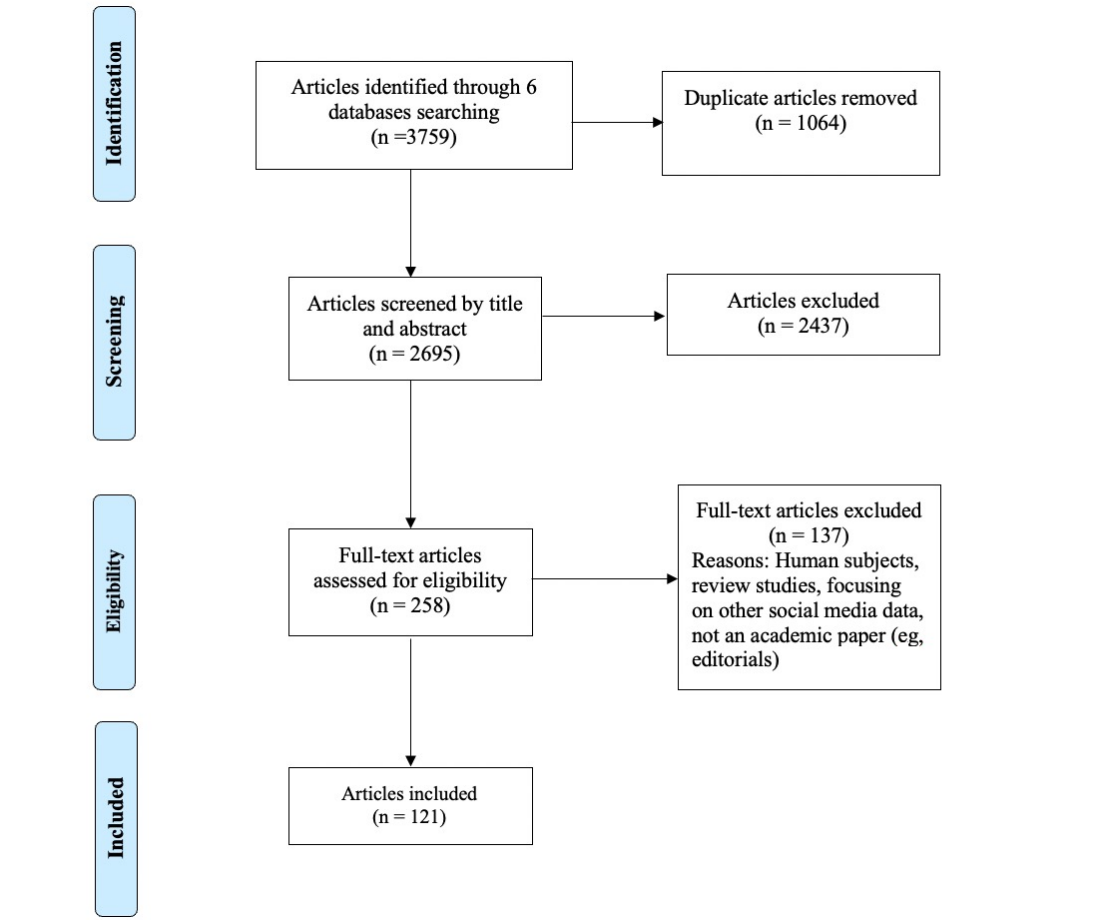
PRISMA (Preferred Reporting Items for Systematic Reviews and Meta-Analyses) flowchart. Selection procedure and search results.

The search retrieved a total of 3759 studies from the 6 selected databases. After removing duplicates (n=1063), 2695 articles were entered into Covidence for abstract screening. To ensure the accuracy of article extraction, 7 research assistants (BZ, QZ, RH, JJ, NL, YP, and ZL) were trained to participate in both abstract and full-text screening processes to extract relevant studies. Each study was independently reviewed by 2 research assistants using the screening software, Covidence. The principal investigator (PI) (JX) resolved all disagreements. Before the screening process began, the PI trained all research assistants on screening guidelines. Following the abstract screening, 258 studies were selected for full-text screening. Of these, 137 studies were excluded in the full-text screening stage, resulting in a final sample of 121 articles that met the inclusion criteria. A PRISMA (Preferred Reporting Items for Systematic Reviews and Meta-Analyses) flowchart, shown in Figure 1, illustrates the search and screening process. By adhering to a comprehensive screening process, this review aimed to ensure the selection of studies that met the predefined inclusion criteria and to minimize bias in the study selection process.

### Charting the Data

The team developed a charting guideline for extracting essential information from the articles included in the review. The charting guideline was organized into several main categories, including publication year, study objectives, theoretical or conceptual framework, use of Twitter data in the study, use of Twitter metadata, data collection methods, sample size, data analysis strategy, key findings, ethical considerations, and study limitations. Seven research assistants participated in the data charting process. Prior to the charting launch, all research assistants completed a charting exercise by independently reviewing the same 4 selected articles to familiarize themselves with the charting guideline and identify any areas that needed further clarification. The research team met to debrief the charting exercise and refine the charting guideline accordingly. The intercoder reliability score was excellent (Cohen κ >90%). The PI then assigned each article to 2 research assistants for independent charting. The charting results from all research assistants were compiled into an Excel sheet, and any discrepancies were resolved by the PI. The team spent 3 months completing the screening and another 3 months for data extraction.

### Collating, Summarizing, and Reporting the Results

Using a scoping review design, this study aimed to provide a comprehensive knowledge synthesis that maps the current state of sexual violence studies using Twitter data and identifies gaps for future research. As per the scoping review framework [27], the quality of the selected studies was not appraised in this study.

## Results

### Overview

In this study, a total of 121 articles were reviewed out of 3759 studies initially identified through the search. We reviewed and described the current state of sexual violence research using Twitter data, including publication year, publication sources (ie, journal or conference), research topic, research methods, data collection methods, sample size, timeframe of tweets collected, data analysis strategy, and institutional review board procedures for using Twitter data in sexual violence research. To characterize Twitter use as a data source in these articles, we developed a codebook to define the taxonomy for different metadata features that can be extracted from Twitter, including Twitter use (eg, content/text, social network, and sentiment/emotions), Twitter functions (eg, #hashtags, URLs, and geotags/locations), and Twitter users (eg, age, gender, and occupation). We also extracted information about the results and discussions of the assessed studies but did not report them in this article.

### Current State of Sexual Violence Research Using Twitter Data

We extracted information about the publication year, publication source (ie, journal or conference), and research topics. Discussions related to institutional review boards or ethics reviews were also extracted.

#### Publication Year

Multimedia Appendix 2 shows the number of articles published each year till April 2022. The review found that most articles were recent, with 68.6% (83/121) published after 2018. However, the earliest study in this review, which examined the information dissemination about the 2012 New Delhi gang rape protest [30], was published in 2013. The findings suggest that using Twitter data in sexual violence research is a relatively recent development, with a considerable increase in publications in recent years.

#### Type of Journal or Conference

Findings showed that journals were the most common publication type for the reviewed articles, with 73.6% (89/121) of the articles published in journals. The reviewed articles most favored the following journals: *Journal of Interpersonal Violence*, *Journal of Medical Internet Research*, *Feminist Media Studies*, *Violence and Gender*, and *Journal of Community Psychology*. The remaining journal articles were published across various disciplines. A total of 28 of 121 (23.1%) articles were published at conferences. The findings suggest a growing interest in using Twitter data for sexual violence research across different fields.

#### Research Topics

Regarding research topics, the reviewed articles used 511 different hashtags to reference their research focus. The top 10 most commonly used hashtags were #MeToo (n=71), #NotOkay (n=21), #WhyIStayed (n=18), #WhyILeft (n=12), #WhyIDidntReport (n=9), #YesAllWomen (n=8), #BeenRapedNeverReported (n=5), #BlackLivesMatter (n=5), and #domesticviolence (n=5). However, 451 hashtags only appeared once in the reviewed articles. The findings indicate that most articles focused on similar themes surrounding focusing events or social movements in the society.

#### Research Objectives

This scoping review examined the current state of using Twitter data for sexual violence research, with a focus on publication year, publication source (ie, journal or conference), research objectives, and ethical considerations surrounding using Twitter data. We identified 7 main objectives after reviewing and summarizing the stated research objectives, including (1) exploring online disclosures and public opinions of sexual violence victimization [20,31]; (2) analyzing Twitter activities and discussions about focusing events or cases related to sexual violence, such as “Wolf pack,” “Hawthron case,” [32] “New Delhi Gangrape,” “Ray Rice,” and “Janay Rice” [23]; (3) investigating cultural perceptions of sexual assault [33]; (4) building tools to capture offensive and abusive language on Twitter [34]; (5) using Twitter as a tool to set public agenda and influence policies related to sexual violence, for example, Clark and Evans [35] examined how factors (ie, gender, partisanship, and ideology) influence Congress members’ tweet activities about the #MeToo movement; (6) building and testing algorithms to categorize tweets containing harassment [36,37]; and (7) examining public discourses under popular sexual assault–related hashtags (ie, #whyistatyed and #whyididn’treport) [20] or with key terms of “domestic violence” [38]. When using Twitter as a data source, most studies focused on analyzing tweets to examine sexual assault events in the society, such as assessing the reactions of Twitter users as supportive or detractive, as well as exploring the public discourse and personal revelation surrounding sexual violence. These studies used Twitter as a lens to examine high-profile cases of sexual violence and the reasons for staying in abusive relationships.

#### Ethical Discussions

Out of the 121 reviewed articles, only 2 indicated that they had obtained ethics approval for using Twitter data for sexual violence research from an Institutional Review Board [21,39]. Eleven studies mentioned that Twitter’s terms of service were considered consent for using Twitter data or discussed the exemption of tweets, as publicly available information, from ethical reviews. Since we did not include articles that used Twitter as a platform to recruit participants, traditional written consent with study participants did not apply to this scoping review. The ethical implications of using Twitter data for sexual violence research were also explored in this scoping review. The findings highlight the need for further attention to ethical considerations when using Twitter data for sexual violence research.

### Taxonomy of Using Twitter Data

We employed a predefined taxonomy codebook to categorize how Twitter data were used as a data source in the reviewed articles. The taxonomy included the following 5 categories: (1) content/text, (2) sentiment/emotions, (3) images/photos, (4) engagement (ie, campaigns and nonprofits), and (5) social networks (Table 1). Among the 121 reviewed articles, 112 (92.5%) analyzed the content/text of the tweets, and 31 (25.6%) focused on the sentiment expressed in the tweets (eg, positive or negative emotions). A small number of articles used Twitter to engage people for campaigns (n=5), and 2 studies involved image analysis in tweets. Furthermore, 14.9% (18/121) of the studies analyzed the relationships between networks of Twitter accounts, such as follower/following ratios, and other network characteristics. It is worth noting that studies that used Twitter as a platform to recruit participants or as an intervention were excluded from this review as we focused solely on using Twitter as a data source in this review.

**Table 1:** Twitter data usage taxonomy.

Taxonomy (use of Twitter data)	Description	Article frequency (N=121), n (%)
Content/text	Analysis of the tweets’ text for themes	112 (92.5)
Sentiment/emotions	Analysis of the tweets’ emotions	31 (25.6)
Images/photos	Analysis of the tweets’ associated images	2 (1.6)
Engagement (ie, campaigns and nonprofits)	Analysis of using Twitter to engage people for a campaign or use by nongovernmental organizations	5 (3.3)
Social networks	Analysis of the relationships between users/accounts	18 (14.9)

### Twitter Metadata

In the 121 reviewed articles, hashtags were the most common Twitter metadata feature used (103/121, 85.1%), followed by tweet time and date (52/121, 43.0%), retweets (46/121, 38.0%), replies (ie, @mention; 37/121, 30.6%), URLs (22/121,18.2%), and geotags (25/121, 20.7%) (Table 2). Among the 121 articles, the majority (89/121, 73.5%) used multiple hashtags. For example, Rai et al [40] mentioned a total of 53 hashtags in their article. Only a minority of the articles (18/121, 14.9%) used a single hashtag, and a smaller portion (14/121, 11.6%) did not include any hashtags at all.

To determine metadata inclusion, we coded articles with 1 if they explicitly mentioned the 140-character limitation for tweets and 0 if not. Similarly, for URLs and hashtags, articles were coded as 1 if the URLs were explicitly shown as preserved in the data and 0 if the URLs were explicitly stated as cleaned from the data. The image/photo feature was coded as 1 for articles that included tweet images in the data set, regardless of their inclusion in the analysis. Few studies analyzed the demographic characteristics of Twitter users, such as gender (31/121, 25.6%), race (17/121, 14.0%), age (14/121, 11.6%), and occupation (13/121, 10.7%) (Table 3).

**Table 2:** The use of Twitter metadata in the reviewed articles (2013-2022).

Metadata about tweets	Coding	Number of articles (N=121), n (%)
140-character limitation	Coded as 1 if the 140-character limitation of the tweets is explicitly mentioned in the article.	41 (33.9)
#Hashtags	Coded as 1 if the hashtags are explicitly shown in the data set.	103 (85.1)
URLs	Coded as 1 if the URLs are explicitly shown in the data set.	22 (18.2)
Geotags/locations	Coded as 1 if the geotags/locations are explicitly shown in the data set.	25 (20.7)
Retweets	Coded as 1 if the retweets are explicitly shown in the data set.	46 (38.0)
Images/photos	Coded as 1 if the images or photos are stated in the data set.	13 (10.7)
Tweet time and date	Coded as 1 if the time and date of the tweets are indicated in the data set.	52 (43.0)
Favorites	Coded as 1 if the favorites of the tweets are explicitly mentioned in the article.	12 (9.9)
Replies (@mention)	Coded as 1 if the replies to the tweets are explicitly mentioned in the article.	37 (30.6)
Others	N/A^a^	22 (18.2)

^a^N/A: not applicable.

**Table 3:** Metadata about Twitter users.

Metadata about users	Number of articles (N=121), n (%)
Followers	16 (13.2)
Followings	7 (5.8)
Age	14 (11.6)
Gender	31 (25.6)
Race	17 (14.0)
Occupation	13 (10.7)
Others	21 (17.4)

### Research Methods

#### Data Collection Methods

The application programming interface (API) was used to collect tweets in more than a third of the reviewed studies (51/121, 42.1%), followed by Twitter’s advanced search function (17/121, 14.0%) and NVivo Addition NCapture (12/121, 9.9%). The remaining studies used various tools such as Topsy, GetOldTweets, Meltwater, Netlytic.org, NodeXL, Radian6, Discover Text, LexisNexis, and R. However, we found that 8 articles, such as the study by More and Francis [41], did not provide specific details regarding their data collection methods, stating that the “data is collected and scraped online on Twitter.” Of the 121 reviewed articles, 28 used hashtags as keywords to collect data. Among these, 5 articles used multiple hashtags, with 1 article using 6 keywords [42]. The remaining 23 articles only employed a single hashtag as a keyword to collect data.

#### Sample Size and Timeframe of Tweets Collected

The range of sample sizes varied greatly from 93 tweets [17] to 32 million tweets [43]. Multimedia Appendix 2 shows the distribution of the sample sizes collected and analyzed across the reviewed articles. Eight articles were excluded due to a lack of sample size reporting [44].

About 21% (25/121, 20.7%) of the reviewed articles collected tweets within 7 days, while one-third of the articles (41/121, 33.9%) collected tweets over an 8- to 31-day period. About 30% (36/121, 29.8%) of the articles collected tweets for longer than 1 month (31 days) but less than 1 year (365 days), and 10 articles collected tweets for over 12 months.

To obtain the average number of tweets collected per day, the sample size was divided by the number of days collected. Among the articles, 30 collected fewer than 100 tweets daily, 31 collected between 100 and 999 tweets daily, 26 collected between 1000 and 9999 tweets daily, 15 collected between 10,000 and 99,999 tweets daily, and 3 collected more than 100,000 tweets daily.

In terms of the year of tweets collected, the highest number of tweets were posted in 2017 (43/180, 23.9%), with more than half of the tweets posted between 2016 and 2018 (Multimedia Appendix 2).

#### Data Analysis Strategy

Our review revealed 2 main aspects, namely coding strategies and analysis methods. Most studies (n=76) relied on manual coding of tweets, while 13 studies used a combination of manual coding and machine learning coding strategies. For example, Chowdhury et al [45] developed a manual annotation to identify Twitter disclosures of sexual violence.

Regarding the analysis methods employed in the reviewed articles, we identified 6 main approaches: qualitative analysis, sentiment analysis, supervised machine learning, unsupervised machine learning, social network analysis, and quantitative analysis. These categories extend the previously discussed taxonomies of Twitter data usage and coding strategies. A combined approach of qualitative and unsupervised machine learning strategies was reported by 5 studies [46-48]. The most commonly used approach for analyzing Twitter data was a qualitative one, such as thematic analysis, which was employed by 94 studies [49,50]. Machine learning techniques were used in 45 studies to analyze Twitter data, with 21 using supervised learning [51] and 15 using unsupervised learning [52]. Sentiment analysis was performed in 25 of the reviewed studies [23,51], while 12 adopted social network analysis [24,53]. Additionally, 10 studies employed other forms of quantitative analysis [54,55], and 1 study presented their developed Twitter data corpus without mentioning their data analysis approach [34].

Regarding the software and programming languages used for data analysis, Python was found to be the most commonly used language (n=19) [9,15,25,56], followed by NVivo, a qualitative software used in 10 studies for organizing and analyzing tweets [57,58]. R software was also popular, with 7 studies using it for data analysis [59,60]. The text analysis program Linguistic Inquiry and Word Count (LIWC) was used in 6 studies [55,61], and Dedoose was used for analyzing tweet text in 3 studies [21,48]. NodeXL, an Excel add-in, was used for social network analysis in 2 studies [14,30]. Other software mentioned in the reviewed articles included AntConc [62], Gephi [53], InfraNodus [63], The Meaning Extraction Helper [33], SÉANCE [24], Stata [64], and Meltwater (previously Sysomos) [65]. However, we found that 56 of 121 (46.3%) articles did not specify the software, programming language, or app used to analyze tweet text or Twitter metadata. 

## Discussion

### Principal Findings

Over the past decade, researchers have shown an increased interest in using Twitter data to conduct sexual violence research. This scoping review included 121 articles that were published between 2006 and 2022, with an increase in the number of publications since 2013, peaking in 2019 and 2020. Almost 75% (89/121, 73.6%) of the reviewed articles were published in peer-reviewed journals across different disciplines. This review has a couple of main findings. First, it described the current use of Twitter data in sexual violence. Second, it presented a taxonomy outlining how Twitter data are used in this field, including analyzing content/text, sentiment/emotions, images/photos, engagement (ie, campaigns and nonprofits), and social networks. Third, it described and evaluated the methodology applied in the reviewed studies.

### Taxonomy of Using Twitter Data

This review developed a taxonomy to describe the use of Twitter data in sexual violence research, which included the following 5 categories: (1) content/text, (2) sentiment/emotions, (3) images/photos, (4) engagement (ie, campaigns and nonprofits), and (5) social networks. More than 90% (112/121, 92.5%) of the reviewed studies used Twitter text for analysis. For example, Aurrekoetxea-Casaus [66] examined tweet discussions around the hashtags #lamanada [#thewolfpack] in Spain, using qualitative thematic and content analysis. The author found that Twitter is used as a “loudspeaker” to spread myths about sexual violence rather than to provide explicit support for victims. Nutbeam and Mereish [67] analyzed 508 tweets containing the hashtag #MeToo with qualitative content analysis to explore negative attitudes of condemnation or ambivalence toward sexual assault abusers in the MeToo movement. They found novel motives to explain the accusations of the MeToo movement and its accusers, such as wealth, malice, political gain, revenge, and fame, which are not frequently addressed in the literature. Another method of analyzing Twitter-generated data is to assess tweets for public positive or negative reactions [68]. For example, Stevens et al [69] studied the uncivil reactions to sexual assault on Twitter and Reddit by measuring the linguistic characteristics of the news media, such as negative emotions. They found a strong correlation between disagreement language and incivility of Twitter posts, and observed that increased negative emotions reduced identity attacks.

The technical paper by Kumar and Aggarwal [70] reported the methods of extracting sentiments from tweets and calculating the percentages of positive, negative, and neutral tweets to assess the safety of various cities for women. For instance, their study found that Chennai is the safest city for women, while Delhi is the least safe based on the sampled tweets. However, it is important to note that sentiment analysis is cross-sectional, meaning that results are influenced by the date and location of the tweets and are subject to changes in public opinion due to trending news or events in the year.

Social network analysis, which examines the relationship and interactions between Twitter users [65], was employed in 18 studies. Sim et al [71] in Singapore collaborated with the Association of Women for Action and Research (AWARE) to examine how they can reach out to the audience and drive greater awareness of sexual harassment on social media. To achieve this, they built an analytics pipeline that performs digital social listening on conversations about sexual harassment. They identified a network of top users whose tweets were most frequently liked and top users who acted as brokers in the network. They suggested that the AWARE can engage these users to spread information and increase their outreach efforts.

The smallest category of studies in our review focused on the analysis of Twitter images (n=2) or engaged people in campaigns (n=5). Hassan et al [18] examined the Face++ API to analyze Twitter users’ profile pictures to identify their gender and age. The study by Navarro and Coromina [12] examined the public reaction on Twitter following the judicial sentence of the *La Manada* case in Spain on April 26, 2018. They analyzed the images in the posts and found that the images broadened the layers of meaning in the public discourse on Twitter. Despite being a small category, the analysis of Twitter images and campaigns can provide valuable insights into the public’s attitudes and perceptions related to sexual violence.

### Research Methods

This review represents sexual violence research that uses Twitter data, with an estimated 79.6 million tweets analyzed. The most common approach used to access Twitter data was Twitter’s API or Twitter’s advanced search function. Other platforms, such as Discover Text, provided access to the entire collected tweet data set at a cost. Twitter’s API has several advantages for academic research, including free access and the ability to retrieve up to 10 million tweets per month using advanced search operators [72]. Notably, there was variation in sample sizes among the reviewed studies, with some researchers claiming a small sample size (eg, n=300) as a limitation that prevented them from making generalized claims when not adopting a big data approach [56].

A previous review stated that 38 data features could be extracted for each tweet, but Twitter metadata was not fully used in the studies [68]. In this review, we found that many data features were still not included in the analyzed studies. In particular, there was a lack of analysis on metadata about Twitter users, such as their demographic characteristics like gender, age, and occupation. One reason for this underuse of Twitter metadata is the limitations of data collection tools. For example, NVivo Addition NCapture does not collect demographic information [20]. In addition, Twitter users often selectively share information in their public social media profiles, which can lead to a considerable number of missing values in the metadata.

Additionally, the passive nature of data analysis can only retrieve demographic information if it is available on the users’ profiles [73]. However, some studies used advanced data science models to classify the gender of the users. For example, Mueller et al [42] used a convolutional neural network model called “NeuralGenderDemographer” to examine demographic features, including gender and racial/ethnic identities, and their intersections. They found that tweets posted by White women were overrepresented in the #MeToo movement, while tweets posted by Black women revealed more criticisms of the unequal treatment in the social justice system.

This review highlights a potential limitation of underutilizing demographic information about Twitter users, which could decontextualize study findings. Understanding the specific communities that contribute to public opinion on Twitter is crucial to fully grasp the social impact of sexual violence on society. However, researchers must balance this with the ethical considerations of protecting Twitter users’ privacy and anonymity, particularly in sensitive topics such as sexual violence. As a result, demographic information, such as gender, race, and location, may be intentionally removed to protect the identity of users. This suggests a research gap and an opportunity to explore ways of better contextualizing social media data while maintaining confidentiality and privacy.

### Review Limitations

There are several limitations in this review that need to be acknowledged. First, we only included articles that used Twitter data (eg, text and photos) to research sexual violence or assault. Studies that used Twitter for intervention or participation recruitment purposes were excluded. Second, the search terms were limited to certain types of sexual violence, such as sexual assault, rape, sexual abuse, sexual harassment, and gender-based violence (Multimedia Appendix 1). Studies focusing on other forms of violence, such as physical abuse, psychological abuse, economic control, and verbal abuse, were excluded from the scoping review. It is worth noting that some of the reviewed studies examined sexual violence as well as other forms of violence such as physical assault. Third, our search strategy did not include key terms, such as “infoveillance” and “infodemiology,” which are important concepts in the field of public health research [74]. Despite these limitations, this review is the first to comprehensively synthesize and evaluate the current use of social media data in sexual violence research, providing valuable insights into the field.

### Future Research: Recommendations for Using Twitter Data in Sexual Violence Research

This scoping review demonstrates the potential of Twitter data as a valuable resource in sexual violence research. This method allows researchers to expand beyond traditional data collection methods like surveys, interviews, and administrative data for sexual violence research. However, it is important to note that gaps exist in the current literature, and a further study is needed to fully understand the potential and limitations of using Twitter as a data source for sexual violence research.

#### Development of Methods for Collecting and Analyzing Twitter Data

This scoping review highlights the need to develop more robust methods for data collection and analysis. Fourteen studies reported that a small sample size restricted the generalizability of their findings [60]. In addition, some studies pointed out problems with the algorithms employed in data processing. For example, Purohit et al [31] found that the LIWC tool required revision in order to effectively process the unconstrained natural language text of Twitter data.

On the other hand, Lowenstein-Barkai [75] suggested that qualitative thematic analysis was crucial for placing quantitative findings within a broader context. To address the limitations of research capacity, future research could benefit from strengthening interdisciplinary collaborations between computer science and sexual violence researchers, with a focus on qualitative interpretations that support solutions to social problems. This could involve exploring specific computational methods such as linguistics and geospatial analysis.

#### Ethical Considerations Surrounding the Use of Twitter Data

The ethical considerations of using Twitter data in sexual violence research have been discussed in a limited number of studies. Only 10.7% (13/121) of the reviewed studies explicitly reported ethical considerations. In these articles, direct interaction with human subjects was not involved, and formal informed consent was not required. To ensure the anonymity of the participants, quotes were modified to avoid reverse identification [76], and traceable quotes were not included [63]. Usher et al [63] demonstrated the use of secure encryption of Twitter data through Meltwater and InfraNodus, minimizing potential harm to participants during data collection and analysis. Despite these efforts, future research should engage in in-depth discussions regarding ethical norms and guidelines to further protect the privacy and confidentiality of participants. For example, research examining the methods of inferring user demographics, such as gender, age, ethnicity, education, and income, from social media posts has gained increasing attention in recent years. This approach is particularly relevant for Twitter research [77]. Unlike traditional survey methods that directly ask participants for demographic information, predicting the demographic characteristics of Twitter users from their posts poses significant challenges [78-80]. Inferring demographic information from Twitter data is often associated with substantial errors and ethical challenges relating to privacy and consent. Such discussions may lead to developing more comprehensive and robust ethical standards for using Twitter data in sexual violence research.

#### Specific Aspects of Sexual Violence on Twitter

Further examination is needed to gain a more comprehensive understanding of particular aspects of sexual violence on Twitter. One crucial area for exploration is the manifestation of intersectionality in tweets. Kachen et al [46] suggested that future research should examine how intersectional differences may affect the semantic meaning and sentiment of tweets. Cultural backgrounds, racialization, socioeconomic status, and other particular experiences of social oppression and privileges may all interact and influence the contents and interactions of tweets related to sexual violence. For example, the hashtag #MeToo, used to discuss sexual violence, may be used in conjunction with other hashtags related to racial justice movements (eg, #BlackLivesMatter) and gender inclusion rights advocacy (eg, #TransRightsAreHumanRights). Exploring some forms of combinations of hashtags in future studies may be one of the practical intersectional approaches that provide a more nuanced and comprehensive understanding of how different social identities and experiences with intersecting privileges and oppressions intersect and shape the experiences of and perspectives on sexual violence. Ultimately, adopting intersectional lenses to collect and analyze social media data contributes to creating more thoughtful and inclusive antiviolence strategies, social programs, and policies.

#### English Language Tweets

Most of the reviewed studies only coded and analyzed English language tweets, leading to concerns about potential bias. Sixteen articles noted the inherent bias in their methodology, as they only included English tweets [81]. Research suggests that “English-only tweets may reflect national hotspot areas in which English is spoken more commonly and thus have higher rates of English language tweets regarding any topic” [39]. The #MeToo hashtag was widely used to collect tweets related to sexual violence in the reviewed studies. However, as a global movement, the stories of the #MeToo movement on social media are understudied when current research only examines English language tweets. To address this limitation, future studies should consider sampling tweets in diverse languages, such as #MeToo or similar hashtags in other languages [82]. Meanwhile, researchers may consider collecting Twitter data along with data from other social media that are more commonly used by individuals who prefer digital communication in a language other than English. This approach would help broaden our knowledge of the global impact of antisexual violence campaigns, such as the #MeToo movement, and provide a more comprehensive understanding of how sexual violence is discussed across diverse cultures and languages in the transnational digital space.

#### Underrepresentation

Using Twitter as a data source for sexual violence research may not provide a representative sample of individuals who have experienced sexual violence. Thirty-two reviewed articles revealed that Twitter users do not represent the general population and only reflect opinions at a time, which do not accurately define the users. It is important to note that not all individuals who have experienced sexual violence use Twitter or disclose their experiences on this platform. Guidry et al [83] noted the potential selection bias of individuals who choose to tweet using the hashtag #WhyIDidntReport. Woo et al [84] mentioned that “we cannot definitively ascertain whether the purposes and attitudes of each Twitter post are parallel with those users’ intentions.” Thirteen articles stated that social media platforms, such as Twitter, are not socially representative and may limit the generalizability of the sample and results. For example, Twitter users tend to be younger in the United States [85]. Tweet posters may be those who feel comfortable discussing sexual assault topics in public or those who are less traumatized when they disclose their own experience. It is important to recognize this limitation and mitigate it by intentionally collecting additional data from social groups whose perspectives and experiences are underrepresented on social media. This could be mitigated through the collection of data from other sources, such as traditional surveys and interviews, and different social media platforms.

### Conclusions

Existing literature shows the potential of using Twitter-based data for sexual violence research, as evidenced by the increasing number of publications after 2018. To contribute to this field, this study reviewed and extracted 121 articles that collectively analyzed about 79.6 million tweets. We described the current state of using Twitter data for sexual violence research, developed a new taxonomy that describes Twitter as a data source, and evaluated the methodologies used. Based on our review, several research recommendations are proposed, such as development of data collection and analysis methods, in-depth discussions about ethical norms, exploration of specific aspects of sexual violence on Twitter, analysis of tweets in multiple languages, and a more inclusive representation of the findings. These recommendations may guide future studies on using Twitter data for sexual violence research.

## Appendix

Multimedia Appendix 1 Search strategies.

Multimedia Appendix 2 Data regarding publication year, sample size, and year of data collection.

## Abbreviations

API: application programming interface
AWARE: Association of Women for Action and Research
LIWC: Linguistic Inquiry and Word Count
PI: principal investigator

## References

[1] Black MC, Basile KC, Breiding MJ, Smith SG, Walters ML, Merrick MT, Chen J, Stevens MR (2011). National Intimate Partner and Sexual Violence Survey: 2010 Summary Report. Centers for Disease Control and Prevention.

[2] Krug EG, Mercy JA, Dahlberg LL, Zwi AB (2002). The world report on violence and health. The Lancet.

[3] Fanslow J, Robinson E (2004). Violence against women in New Zealand: prevalence and health consequences. N Z Med J.

[4] (2014). Violence against women : intimate partner and sexual violence against women : intimate partner and sexual violence have serious short- and long-term physical, mental and sexual and reproductive health problems for survivors : fact sheet. World Health Organization.

[5] Su Y, Xue J, Liu X, Wu P, Chen J, Chen C, Liu T, Gong W, Zhu T (2020). Examining the Impact of COVID-19 Lockdown in Wuhan and Lombardy: A Psycholinguistic Analysis on Weibo and Twitter. Int J Environ Res Public Health.

[6] Xue J, Chen J, Hu R, Chen C, Zheng C, Su Y, Zhu T (2020). Twitter Discussions and Emotions About the COVID-19 Pandemic: Machine Learning Approach. J Med Internet Res.

[7] Xue J, Chen J, Chen C, Zheng C, Li S, Zhu T (2020). Public discourse and sentiment during the COVID 19 pandemic: Using Latent Dirichlet Allocation for topic modeling on Twitter. PLoS One.

[8] Xiang X, Lu X, Halavanau A, Xue J, Sun Y, Lai PHL, Wu Z (2021). Modern Senicide in the Face of a Pandemic: An Examination of Public Discourse and Sentiment About Older Adults and COVID-19 Using Machine Learning. J Gerontol B Psychol Sci Soc Sci.

[9] Xue J, Chen J, Chen C, Hu R, Zhu T (2020). The Hidden Pandemic of Family Violence During COVID-19: Unsupervised Learning of Tweets. J Med Internet Res.

[10] López G, Bogen KW, Meza-Lopez RJ, Nugent NR, Orchowski LM (2022). #DomesticViolence During the COVID-19 Global Pandemic: An Analysis of Public Commentary via Twitter. Digit Health.

[11] Bogen KW, Bleiweiss K, Orchowski LM (2019). Sexual violence is #NotOkay: Social reactions to disclosures of sexual victimization on twitter. Psychology of Violence.

[12] Navarro C, Coromina Ò (2020). Discussion and mediation of social outrage on Twitter: The reaction to the judicial sentence of "La Manada". Communication & Society.

[13] Zimmerman T (2017). #Intersectionality: T e Fourth Wave Feminist Twitter Community. Atlantis.

[14] Puente SN, Maceiras SD, Romero DF (2019). Twitter Activism and Ethical Witnessing: Possibilities and Challenges of Feminist Politics Against Gender-Based Violence. Social Science Computer Review.

[15] Xue J (2018). Agenda-Setting for Intimate Partner Violence: Exploring the Role of Social Media United States - Based Twitter. ProQuest.

[16] Starzynski LL, Ullman SE, Filipas HH, Townsend SM (2005). Correlates of Women's Sexual Assault Disclosure to Informal and Formal Support Sources. violence vict.

[17] Alaggia R, Wang S (2020). "I never told anyone until the #metoo movement": What can we learn from sexual abuse and sexual assault disclosures made through social media?. Child Abuse Negl.

[18] Hassan N, Mandal MK, Bhuiyan M, Moitra A, Ahmed SI (2019). Can women break the glass ceiling?: an analysis of #MeToo hashtagged posts on Twitter. ASONAM '19: Proceedings of the 2019 IEEE/ACM International Conference on Advances in Social Networks Analysis and Mining.

[19] Bogen KW, Millman C, Huntington F, Orchowski LM (2018). A Qualitative Analysis of Disclosing Sexual Victimization by #NotOkay During the 2016 Presidential Election. Violence and Gender.

[20] Bogen KW, Mulla MMM, Haikalis M, Orchowski LM (2022). Sexual Victimization Among Men: A Qualitative Analysis of the Twitter Hashtag #UsToo. J Interpers Violence.

[21] Storer HL, Rodriguez M, Franklin R (2021). "Leaving Was a Process, Not an Event": The Lived Experience of Dating and Domestic Violence in 140 Characters. J Interpers Violence.

[22] Cravens JD, Whiting JB, Aamar RO (2015). Why I Stayed/Left: An Analysis of Voices of Intimate Partner Violence on Social Media. Contemp Fam Ther.

[23] Davis JL, Love TP (2018). Women Who Stay: A Morality Work Perspective. Social Problems.

[24] Eiler BA, Al-Kire R, Doyle PC, Wayment HA (2019). Power and Trust Dynamics of Sexual Violence: A Textual Analysis of Nassar Victim Impact Statements and #MeToo Disclosures on Twitter. Journal of Clinical Sport Psychology.

[25] Xue J, Macropol K, Jia Y, Zhu T, Gelles RJ (2019). Harnessing big data for social justice: An exploration of violence against women‐related conversations on Twitter. Human Behav and Emerg Tech.

[26] Ahrens CE (2006). Being silenced: the impact of negative social reactions on the disclosure of rape. Am J Community Psychol.

[27] Arksey H, O'Malley L (2005). Scoping studies: towards a methodological framework. International Journal of Social Research Methodology.

[28] McGowan J, Sampson M, Salzwedel DM, Cogo E, Foerster V, Lefebvre C (2016). PRESS Peer Review of Electronic Search Strategies: 2015 Guideline Statement. J Clin Epidemiol.

[29] Basile KC, Clayton HB, Rostad WL, Leemis RW (2020). Sexual Violence Victimization of Youth and Health Risk Behaviors. Am J Prev Med.

[30] Ahmed S, Jaidka K, Urs SR, Na JC, Buchanan G (2013). The Common Man: An Examination of Content Creation and Information Dissemination on Twitter during the 2012 New Delhi Gang-Rape Protest. Digital Libraries: Social Media and Community Networks. ICADL 2013. Lecture Notes in Computer Science, vol 8279.

[31] Purohit H, Banerjee T, Hampton A, Shalin VL, Bhandutia N, Sheth AP (2015). Gender-Based Violence in 140 Characters or Fewer: A #BigData Case Study of Twitter. arXiv.

[32] Waterhouse-Watson D (2019). “I Would Hate To See Our Good Name Tarnished”: Twitter Users Respond to Sexual Assault in Football. Journal of Australian Studies.

[33] Ikizer EG, Ramírez-Esparza N, Boyd RL (2018). #sendeanlat (#tellyourstory): Text Analyses of Tweets About Sexual Assault Experiences. Sex Res Soc Policy.

[34] Rezvan M, Shekarpour S, Balasuriya L, Thirunarayan K, Shalin VL, Sheth A (2018). A Quality Type-aware Annotated Corpus and Lexicon for Harassment Research. WebSci '18: Proceedings of the 10th ACM Conference on Web Science.

[35] Clark JH, Evans HK (2019). Let’s Talk about Sex: Examining the Factors Influencing Congressional Response to #MeToo on Twitter. APSC.

[36] Saeidi M, da S Sousa SB, Milios E, Zeh N, Berton L, Cellier P, Driessens K (2019). Categorizing Online Harassment on Twitter. Machine Learning and Knowledge Discovery in Databases. ECML PKDD 2019. Communications in Computer and Information Science, vol 1168.

[37] Kumari N, kandukuri P (2019). Dynamic Data Analysis and Decision Making on Twitter Data. IJITEE.

[38] Xue J, Chen J, Gelles R (2019). Using Data Mining Techniques to Examine Domestic Violence Topics on Twitter. Violence and Gender.

[39] Bogen KW, Orchowski LM (2021). A Geospatial Analysis of Disclosure of and Social Reactions to Sexual Victimization on Twitter Using #MeToo. Women & Therapy.

[40] Rai A, Choi YJ, Cho S, Das U, Tamayo J, Menon GM (2021). “#Domestic Violence Isn’t Stopping for Coronavirus …….”: Intimate Partner Violence Conversations on Twitter during the Early Days of the COVID-19 Pandemic. Journal of Evidence-Based Social Work.

[41] More K, Francis F (2021). Analyzing the Impact of Domestic Violence on Social Media using Natural Language Processing.

[42] Mueller A, Wood-Doughty Z, Amir S, Dredze M, Lynn Nobles A (2021). Demographic Representation and Collective Storytelling in the Me Too Twitter Hashtag Activism Movement. Proc ACM Hum Comput Interact.

[43] Prieto Curiel R, Cresci S, Muntean CI, Bishop SR (2020). Crime and its fear in social media. Palgrave Commun.

[44] Maluleke G, Moyer E (2020). “We Have to Ask for Permission to Become”: Young Women’s Voices, Violence, and Mediated Space in South Africa. Signs: Journal of Women in Culture and Society.

[45] Chowdhury AG, Sawhney R, Mathur P, Mahata D, Shah RR (2019). Speak up, Fight Back! Detection of Social Media Disclosures of Sexual Harassment. Proceedings of the 2019 Conference of the North American Chapter of the Association for Computational Linguistics: Student Research Workshop.

[46] Kachen A, Krishen AS, Petrescu M, Gill RD, Peter PC (2020). #MeToo, #MeThree, #MeFour: Twitter as community building across academic and corporate institutions. Psychology & Marketing.

[47] Manikonda L, Beigi G, Liu H, Kambhampati S (2018). Twitter for Sparking a Movement, Reddit for Sharing the Moment: #metoo through the Lens of Social Media. arXiv.

[48] Rodriguez MY, Storer H (2019). A computational social science perspective on qualitative data exploration: Using topic models for the descriptive analysis of social media data. Journal of Technology in Human Services.

[49] Dragiewicz M, Burgess J (2016). Canadian Journal of Women and the Law.

[50] Subramanian R, Weare A (2020). #notokay: Challenging sexual violence through digital health activism. Critical Public Health.

[51] Kuna C, Bai MR, Sreedevi MJ, Shantha DG (2020). Me Too Movement Sentiment Analysis. International Journal of Advanced Science and Technology.

[52] Baik JM, Nyein TH, Modrek S (2022). Social Media Activism and Convergence in Tweet Topics After the Initial #MeToo Movement for Two Distinct Groups of Twitter Users. J Interpers Violence.

[53] Trott V (2020). Networked feminism: counterpublics and the intersectional issues of #MeToo. Feminist Media Studies.

[54] Baer P (2017). Muted Groups and Public Discourse: The Web of Sexual Violence and Social Media. ProQuest.

[55] ElSherief M, Belding E, Nguyen D (2017). #NotOkay: Understanding Gender-Based Violence in Social Media. ICWSM.

[56] Mendes K, Keller J, Ringrose J (2018). Digitized narratives of sexual violence: Making sexual violence felt and known through digital disclosures. New Media & Society.

[57] Maas MK, McCauley HL, Bonomi AE, Leija SG (2018). "I Was Grabbed by My Pussy and Its #NotOkay": A Twitter Backlash Against Donald Trump's Degrading Commentary. Violence Against Women.

[58] McCauley HL, Bonomi AE, Maas MK, Bogen KW, O'Malley TL (2018). #MaybeHeDoesntHitYou: Social Media Underscore the Realities of Intimate Partner Violence. J Womens Health (Larchmt).

[59] Modrek S, Chakalov B (2019). The #MeToo Movement in the United States: Text Analysis of Early Twitter Conversations. J Med Internet Res.

[60] Dragotto F, Giomi E, Melchiorre SM (2020). Putting women back in their place. Reflections on slut-shaming, the case Asia Argento and Twitter in Italy. International Review of Sociology.

[61] Ahmed S, Jaidka K, Cho J (2016). Tweeting India’s Nirbhaya protest: a study of emotional dynamics in an online social movement. Social Movement Studies.

[62] Hardaker C, McGlashan M (2016). “Real men don’t hate women”: Twitter rape threats and group identity. Journal of Pragmatics.

[63] Usher K, Durkin J, Martin S, Vanderslott S, Vindrola-Padros C, Usher L, Jackson D (2021). Public Sentiment and Discourse on Domestic Violence During the COVID-19 Pandemic in Australia: Analysis of Social Media Posts. J Med Internet Res.

[64] Gurman TA, Nichols C, Greenberg ES (2018). Potential for social media to challenge gender-based violence in India: a quantitative analysis of Twitter use. Gender & Development.

[65] Moeke-Pickering T, Cote-Meek S, Pegoraro A (2018). Understanding the ways missing and murdered Indigenous women are framed and handled by social media users. Media International Australia.

[66] Aurrekoetxea-Casaus M (2020). San fermines #la manada case: An exploratory analysis of social support for victims of sexual violence on Twitter. Computers in Human Behavior.

[67] Nutbeam M, Mereish EH (2022). Negative Attitudes and Beliefs Toward the #MeToo Movement on Twitter. J Interpers Violence.

[68] Sinnenberg L, Buttenheim AM, Padrez K, Mancheno C, Ungar L, Merchant RM (2017). Twitter as a Tool for Health Research: A Systematic Review. Am J Public Health.

[69] Stevens H, Acic I, Taylor LD (2021). Uncivil Reactions to Sexual Assault Online: Linguistic Features of News Reports Predict Discourse Incivility. Cyberpsychol Behav Soc Netw.

[70] Kumar D, Aggarwal S (2019). Analysis of Women Safety in Indian Cities Using Machine Learning on Tweets.

[71] Sim X, Chang ER, Ong YX, Yeo JY, Yan CBS, Choy EWJ, Shim KJ (2020). Digital Social Listening on Conversations About Sexual Harassment.

[72] Twitter Developer API. Twitter.

[73] Hawkins LG, Mullet N, Brown CC, Eggleston D, Gardenhire J (2019). All Survivors Have the Right to Heal: A #Metoomen Content Analysis. Journal of Feminist Family Therapy.

[74] Eysenbach G (2009). Infodemiology and infoveillance: framework for an emerging set of public health informatics methods to analyze search, communication and publication behavior on the Internet. J Med Internet Res.

[75] Lowenstein-Barkai H (2021). #Me(n)Too? Online Social Support Toward Male and Female Survivors of Sexual Victimization. J Interpers Violence.

[76] Ayers JW, Caputi TL, Nebeker C, Dredze M (2018). Don't quote me: reverse identification of research participants in social media studies. NPJ Digit Med.

[77] Preoţiuc-Pietro D, Volkova S, Lampos V, Bachrach Y, Aletras N (2015). Studying User Income through Language, Behaviour and Affect in Social Media. PLoS One.

[78] Mislove A, Lehmann S, Ahn Y, Onnela J, Rosenquist J (2021). Understanding the Demographics of Twitter Users. ICWSM.

[79] Nguyen D, Gravel R, Trieschnigg D, Meder T (2021). "How Old Do You Think I Am?" A Study of Language and Age in Twitter. ICWSM.

[80] Culotta A, Kumar N, Cutler J (2015). Predicting the Demographics of Twitter Users from Website Traffic Data. AAAI.

[81] Li M, Turki N, Izaguirre CR, DeMahy C, Thibodeaux BL, Gage T (2021). Twitter as a tool for social movement: An analysis of feminist activism on social media communities. J Community Psychol.

[82] Drewett C, Oxlad M, Augoustinos M (2021). Breaking the silence on sexual harassment and assault: An analysis of #MeToo tweets. Computers in Human Behavior.

[83] Guidry JPD, Sawyer AN, Carlyle KE, Burton CW (2021). #WhyIDidntReport: Women Speak Out About Sexual Assault on Twitter. J Forensic Nurs.

[84] Woo CW, Brigham MP, Gulotta M (2019). Twitter Talk and Twitter Sharing in Times of Crisis: Exploring Rhetorical Motive and Agenda-Setting in the Ray Rice Scandal. Communication Studies.

[85] Perrin A, Anderson M (2019). Share of U.S. adults using social media, including Facebook, is mostly unchanged since 2018. Pew Research Center.

