# Supplementary material for: Using Twitter-Based Data for Sexual Violence Research: Scoping Review
Source: J Med Internet Res. 2023 May 15;25:e46084. doi: 10.2196/46084 (PMC10227696; doi:10.2196/46084)
Supplement: Multimedia Appendix 1 [file jmir_v25i1e46084_app1.docx]

**Multimedia Appendix 1.** Search strategies.

## **APA PsycInfo (Ovid)**

| # | Searches | Results |
| --- | --- | --- |
| 1 | (Sexual* adj3 (assault* or violence or victim* or abus* or harass* or coerci* or aggress* or perpetrat* or offen* or consen* or assailant* or criminal* or attack*)).tw. | 49208 |
| 2 | (Sex adj3 (assault* or violence or victim* or abus* or harass* or coerci* or aggress* or perpetrat* or offen* or consen* or assailant* or criminal* or attack*)).tw. | 10868 |
| 3 | (rape or rapist*).tw. | 9599 |
| 4 | (consen* adj3 (without or non or "not" or lack or "no")).tw. | 6594 |
| 5 | (consensus or informed consent).tw. | 37268 |
| 6 | 4 not 5 | 1381 |
| 7 | (Violen* adj3 (domestic or women or intimate partner* or gender* or dating)).tw. | 23977 |
| 8 | Pedophil*.tw. | 1641 |
| 9 | exp Rape/ | 5951 |
| 10 | Intimate Partner Violence/ | 11602 |
| 11 | Sexual Abuse/ | 20789 |
| 12 | Domestic Violence/ | 11466 |
| 13 | Dating Violence/ | 1164 |
| 14 | Sex Offenses/ | 10160 |
| 15 | or/1-3,6-14 [sexual assault] | 86022 |
| 16 | twitter*.tw. | 2800 |
| 17 | tweet*.tw. | 1191 |
| 18 | hashtag*.tw. | 281 |
| 19 | Online Social Networks/ | 7704 |
| 20 | Social Media/ | 8229 |
| 21 | or/16-20 [twitter] | 15225 |
| 22 | metoo.tw. | 81 |
| 23 | himtoo.tw. | 0 |
| 24 | (notokay or notok).tw. | 4 |
| 25 | Tellyourstory.tw. | 1 |
| 26 | HowIWillChange.tw. | 1 |
| 27 | Whyididntreport.tw. | 1 |
| 28 | (BelieveWomen or BelieveSurvivors).tw. | 0 |
| 29 | TimesUp.tw. | 4 |
| 30 | WhyIStayed.tw. | 2 |
| 31 | or/22-30 [hashtags] | 87 |
| 32 | (15 and 21) or 31 | 259 |
| 33 | limit 32 to yr="2006 -Current" | 259 |

**Scopus**

(((TITLE-ABS-KEY(consen* W/3 ( without OR non OR "not" OR lack OR "no" )) AND NOT TITLE-ABS-KEY(consensus OR "informed consent" )) OR ((TITLE-ABS-KEY ( ( sexual* W/3 ( assault* OR violen* OR victim* OR abus* OR harass* OR coerci* OR aggress* OR perpetrat* OR offen* OR consen* OR assailant* OR criminal* OR attack* ) ) OR ( sex W/3 ( assault* OR violen* OR victim* OR abus* OR harass* OR coerci* OR aggress* OR perpetrat* OR offen* OR consen* OR assailant* OR criminal* OR attack* ) ) OR rape* OR rapist* OR ( violen* W/3 ( domestic OR women OR "intimate partner*" OR gender* OR dating ) ) OR pedophil* )))) AND (TITLE-ABS-KEY(twitter* OR tweet* OR hashtag*))) OR (TITLE-ABS-KEY(Metoo OR Himtoo OR Notokay OR Notok OR Tellyourstory OR HowIWillChange OR Whyididntreport OR BelieveWomen OR BelieveSurvivors OR TimesUp OR WhyIStayed)) AND ( LIMIT-TO ( PUBYEAR,2020) OR LIMIT-TO ( PUBYEAR,2019) OR LIMIT-TO ( PUBYEAR,2018) OR LIMIT-TO ( PUBYEAR,2017) OR LIMIT-TO ( PUBYEAR,2016) OR LIMIT-TO ( PUBYEAR,2015) OR LIMIT-TO ( PUBYEAR,2014) OR LIMIT-TO ( PUBYEAR,2013) OR LIMIT-TO ( PUBYEAR,2012) OR LIMIT-TO ( PUBYEAR,2011) OR LIMIT-TO ( PUBYEAR,2010) OR LIMIT-TO ( PUBYEAR,2009) OR LIMIT-TO ( PUBYEAR,2006) )

**PubMed**

(((“Sex offenses” OR “Sexual Harassment” OR “Spouse Abuse” OR “Domestic Violence” OR Rape OR “Intimate Partner Violence” OR “Gender-Based Violence” OR “Child Abuse, Sexual” OR Pedophilia [MeSH Terms]) OR ((Sex [Title/Abstract] OR Sexual*[Title/Abstract]) AND (assault*[Title/Abstract] OR violen*[Title/Abstract] OR victim*[Title/Abstract] OR abus*[Title/Abstract] OR harass*[Title/Abstract] OR coerci*[Title/Abstract] OR aggress*[Title/Abstract] OR perpetrat*[Title/Abstract] OR offen*[Title/Abstract] OR consen*[Title/Abstract] OR assailant*[Title/Abstract] OR criminal*[Title/Abstract] OR attack*[Title/Abstract])) OR ((violen*[Title/Abstract]) AND (domestic[Title/Abstract] OR women[Title/Abstract] OR "intimate partner*"[Title/Abstract] OR gender*[Title/Abstract] OR dating[Title/Abstract])) OR (rape*[Title/Abstract] OR rapist*[Title/Abstract] OR pedophil*[Title/Abstract]) OR ((consen*[Title/Abstract]) NOT (consensus[Title/Abstract] OR “informed consent”[Title/Abstract]))) AND (( “Social Media”[MeSH Terms]) OR (twitter*[Title/Abstract] OR tweet*[Title/Abstract] OR hashtag*[Title/Abstract]))) OR ((Metoo[Title/Abstract] OR Himtoo[Title/Abstract] OR Notokay[Title/Abstract] OR Notok[Title/Abstract] OR Tellyourstory[Title/Abstract] OR HowIWillChange[Title/Abstract] OR Whyididntreport[Title/Abstract] OR BelieveWomen[Title/Abstract] OR BelieveSurvivors[Title/Abstract] OR TimesUp[Title/Abstract] OR WhyIStayed[Title/Abstract]))

**International Bibliography of Social Sciences (IBSS) (ProQuest)**

(( MAINSUBJECT(Rape OR “Date rape” OR “Serial rape” OR “Statutory rape” OR “Sex crimes” OR “Sexual harassment” OR “Domestic violence” OR “Sex offenders” OR Pedophilia OR “Child abuse & neglect”) OR TIAB((Sexual* N/3 (assault* OR violen* OR victim* OR abus* OR harass* OR coerci* OR aggress* OR perpetrat* OR offen* OR consen* OR assailant* OR criminal* OR attack*)) OR (Sex N/3 (assault* OR violen* OR victim* OR abus* OR harass* OR coerci* OR aggress* OR perpetrat* OR offen* or consen* OR assailant* OR criminal* OR attack*)) OR rape* OR rapist* OR  (violen* N/3 (domestic OR women OR "intimate partner*" OR gender* or dating))  OR Pedophil*) OR TIAB((consen* N/3 (without  OR  non  OR  "not"  OR  lack  OR  "no" )) NOT (consensus  OR  "informed consent")) ) AND ( MAINSUBJECT(“Social networks”) OR TIAB(twitter* OR tweet* OR hashtag*) )) OR TIAB(Metoo OR Himtoo OR Notokay OR Notok OR Tellyourstory OR HowIWillChange OR Whyididntreport OR BelieveWomen OR BelieveSurvivors OR TimesUp OR WhyIStayed)

**Criminal Justice Abstracts (EBSCO)**

| # | Content |  |
| --- | --- | --- |
| S5 | S3 OR S4 |  |
| S4 | S1 AND S2 |  |
| S3 | TI ( (Metoo OR Himtoo OR Notokay OR Notok OR Tellyourstory OR HowIWillChange OR Whyididntreport OR BelieveWomen OR BelieveSurvivors OR TimesUp OR WhyIStayed) ) OR AB ( (Metoo OR Himtoo OR Notokay OR Notok OR Tellyourstory OR HowIWillChange OR Whyididntreport OR BelieveWomen OR BelieveSurvivors OR TimesUp OR WhyIStayed) ) OR KW ( (Metoo OR Himtoo OR Notokay OR Notok OR Tellyourstory OR HowIWillChange OR Whyididntreport OR BelieveWomen OR BelieveSurvivors OR TimesUp OR WhyIStayed) ) OR SU ("METOO movement") |  |
| S2 | TI ( (twitter* OR tweet* OR hashtag*) ) OR AB ( (twitter* OR tweet* OR hashtag*) ) OR KW ( (twitter* OR tweet* OR hashtag*) ) OR SU ( (twitter OR "social media") ) |  |
| S1 | TI ( (Sexual* N3 (assault* OR violen* OR victim* OR abus* OR harass* OR coerci* OR aggress* OR perpetrat* OR offen* OR consen* OR assailant* OR criminal* OR attack*)) OR (Sex N3 (assault* OR violen* OR victim* OR abus* OR harass* OR coerci* OR aggress* OR perpetrat* OR offen* or consen* OR assailant* OR criminal* OR attack*)) OR rape* OR rapist* OR (violen* N3 (domestic OR women OR "intimate partner*" OR gender* or dating)) OR Pedophil*OR (( consen* N3 ( without OR non OR "not" OR lack OR "no" ) ) NOT ( consensus OR "informed consent" ) ) ) OR AB ( (Sexual* N3 (assault* OR violen* OR victim* OR abus* OR harass* OR coerci* OR aggress* OR perpetrat* OR offen* OR consen* OR assailant* OR criminal* OR attack*)) OR (Sex N3 (assault* OR violen* OR victim* OR abus* OR harass* OR coerci* OR aggress* OR perpetrat* OR offen* or consen* OR assailant* OR criminal* OR attack*)) OR rape* OR rapist* OR (violen* N3 (domestic OR women OR "intimate partner*" OR gender* or dating)) OR Pedophil*OR (( consen* N3 ( without OR non OR "not" OR lack OR "no" ) ) NOT ( consensus OR "informed consent" ) ) ) OR KW ( (Sexual* N3 (assault* OR violen* OR victim* OR abus* OR harass* OR coerci* OR aggress* OR perpetrat* OR offen* OR consen* OR assailant* OR criminal* OR attack*)) OR (Sex N3 (assault* OR violen* OR victim* OR abus* OR harass* OR coerci* OR aggress* OR perpetrat* OR offen* or consen* OR assailant* OR criminal* OR attack*)) OR rape* OR rapist* OR (violen* N3 (domestic OR women OR "intimate partner*" OR gender* or dating)) OR Pedophil*OR (( consen* N3 ( without OR non OR "not" OR lack OR "no" ) ) NOT ( consensus OR "informed consent" ) ) ) OR SU ( (“sexual abuse” OR “sexual assault” OR “sexual consent” OR “sexual ethics” OR “sexual harassment” OR “sexual misconduct” OR “domestic violence” OR “Dating violence” OR "intimate partner violence” OR rape OR “child abuse” OR "child sexual abuse” OR pedophilia) ) |  |

**Communications Abstracts (EBSCO)**

| # | Content |  |
| --- | --- | --- |
| S5 | S3 OR S4 |  |
| S4 | S1 AND S2 |  |
| S3 | TI ( (Metoo OR Himtoo OR Notokay OR Notok OR Tellyourstory OR HowIWillChange OR Whyididntreport OR BelieveWomen OR BelieveSurvivors OR TimesUp OR WhyIStayed) ) OR AB ( (Metoo OR Himtoo OR Notokay OR Notok OR Tellyourstory OR HowIWillChange OR Whyididntreport OR BelieveWomen OR BelieveSurvivors OR TimesUp OR WhyIStayed) ) OR KW ( (Metoo OR Himtoo OR Notokay OR Notok OR Tellyourstory OR HowIWillChange OR Whyididntreport OR BelieveWomen OR BelieveSurvivors OR TimesUp OR WhyIStayed) ) OR SU ("METOO movement") |  |
| S2 | TI ( (twitter* OR tweet* OR hashtag*) ) OR AB ( (twitter* OR tweet* OR hashtag*) ) OR KW ( (twitter* OR tweet* OR hashtag*) ) OR SU ( (twitter OR "social media") ) |  |
| S1 | TI ( (Sexual* N3 (assault* OR violen* OR victim* OR abus* OR harass* OR coerci* OR aggress* OR perpetrat* OR offen* OR consen* OR assailant* OR criminal* OR attack*)) OR (Sex N3 (assault* OR violen* OR victim* OR abus* OR harass* OR coerci* OR aggress* OR perpetrat* OR offen* or consen* OR assailant* OR criminal* OR attack*)) OR rape* OR rapist* OR (violen* N3 (domestic OR women OR "intimate partner*" OR gender* or dating)) OR Pedophil*OR (( consen* N3 ( without OR non OR "not" OR lack OR "no" ) ) NOT ( consensus OR "informed consent" ) ) ) OR AB ( (Sexual* N3 (assault* OR violen* OR victim* OR abus* OR harass* OR coerci* OR aggress* OR perpetrat* OR offen* OR consen* OR assailant* OR criminal* OR attack*)) OR (Sex N3 (assault* OR violen* OR victim* OR abus* OR harass* OR coerci* OR aggress* OR perpetrat* OR offen* or consen* OR assailant* OR criminal* OR attack*)) OR rape* OR rapist* OR (violen* N3 (domestic OR women OR "intimate partner*" OR gender* or dating)) OR Pedophil*OR (( consen* N3 ( without OR non OR "not" OR lack OR "no" ) ) NOT ( consensus OR "informed consent" ) ) ) OR KW ( (Sexual* N3 (assault* OR violen* OR victim* OR abus* OR harass* OR coerci* OR aggress* OR perpetrat* OR offen* OR consen* OR assailant* OR criminal* OR attack*)) OR (Sex N3 (assault* OR violen* OR victim* OR abus* OR harass* OR coerci* OR aggress* OR perpetrat* OR offen* or consen* OR assailant* OR criminal* OR attack*)) OR rape* OR rapist* OR (violen* N3 (domestic OR women OR "intimate partner*" OR gender* or dating)) OR Pedophil*OR (( consen* N3 ( without OR non OR "not" OR lack OR "no" ) ) NOT ( consensus OR "informed consent" ) ) ) OR SU ( (“sexual abuse” OR “sexual assault” OR “sexual consent” OR “sexual ethics” OR “sexual harassment” OR “sexual misconduct” OR “domestic violence” OR “Dating violence” OR "intimate partner violence” OR rape OR “child abuse” OR "child sexual abuse” OR pedophilia) ) |  |
