# Supplementary material for: Using Twitter-Based Data for Sexual Violence Research: Scoping Review
Source: J Med Internet Res. 2023 May 15;25:e46084. doi: 10.2196/46084 (PMC10227696; doi:10.2196/46084)
Supplement: Multimedia Appendix 2 [file jmir_v25i1e46084_app2.docx]

**Multimedia Appendix 2.** Data regarding publication year, sample size, and year of data collection.

| Data | Number of Articles, n (%) |
| --- | --- |
| Year Published | |
| 2013 | 1 (0.8%) |
| 2014 | 1 (0.8%) |
| 2015 | 5 (4.1%) |
| 2016 | 7 (5.8%) |
| 2017 | 6 (5.0%) |
| 2018 | 18 (14.9%) |
| 2019 | 28 (23.1%) |
| 2020 | 28 (23.1%) |
| 2021 | 21 (17.4%) |
| 2022 | 6 (5.0%) |
| Sample Size | |
| 1~999 | 19 (16.8%) |
| 1,000~9,999 | 36 (31.9%) |
| 10,000~99,999 | 32 (28.3%) |
| 100,000~999,999 | 17 (15.0%) |
| 1,000,000~9,999,999 | 6 (5.3%) |
| 10,000,000+ | 3 (2.7%) |
| Years of Data Collection | |
| 2007 | 1 (0.6%) |
| 2008 | 1 (0.6%) |
| 2009 | 2 (1.1%) |
| 2010 | 3 (1.7%) |
| 2011 | 4 (2.2%) |
| 2012 | 8 (4.4%) |
| 2013 | 11 (6.1%) |
| 2014 | 23 (12.8%) |
| 2015 | 13 (7.2%) |
| 2016 | 26 (14.5%) |
| 2017 | 43 (23.9%) |
| 2018 | 28 (15.6%) |
| 2019 | 11 (6.1%) |
| 2020 | 5 (2.8%) |
